# Protein-losing enteropathy in a child with hypoplastic left heart syndrome after Fontan palliation: a case report

**DOI:** 10.1093/omcr/omag145

**Published:** 2026-08-03

**Authors:** Sakeena Saife, Sarah Saife, Momen Zetawi

**Affiliations:** Department of Medicine, Faculty of Medicine and Health Sciences, An-Najah National University, Nablus, Palestine; Department of Medicine, Faculty of Medicine and Health Sciences, An-Najah National University, Nablus, Palestine; Department of Medicine, Faculty of Medicine and Health Sciences, An-Najah National University, Nablus, Palestine

**Keywords:** hypoplastic left heart syndrome, Fontan circulation, protein-losing enteropathy, hypoalbuminemia, congenital heart disease

## Abstract

Protein-losing enteropathy (PLE) is a rare but serious complication of the Fontan procedure in patients with single-ventricle physiology. It results from excessive loss of plasma proteins into the gastrointestinal tract, leading to hypoalbuminemia, edema, and ascites. We report the case of a 10-year-old girl with hypoplastic left heart syndrome who underwent staged surgical palliation including the Norwood procedure, bidirectional Glenn shunt, and extracardiac Fontan completion. She presented with progressive abdominal distension, peripheral edema, and severe hypoalbuminemia (albumin 2.3 g/dl). Echocardiography and cardiac catheterization demonstrated a patent Fontan circuit with normal pulmonary artery pressures and no significant obstruction, although the inferior vena cava pressure was mildly elevated at approximately 16 mmHg. The patient was managed with intravenous albumin replacement, diuretics, anticoagulation, sildenafil, and oral budesonide. This case highlights the diagnostic challenges of PLE and emphasizes the importance of early recognition and multidisciplinary management, even in the absence of significant Fontan circuit obstruction, as elevated systemic venous pressure and lymphatic dysfunction may both contribute to disease development.

**Key Clinical Message:**

Protein-losing enteropathy is a rare but potentially life-threatening complication of Fontan circulation. It should be suspected in patients with a history of Fontan palliation who present with unexplained hypoalbuminemia, edema, or ascites, even when no significant Fontan circuit obstruction is identified, as elevated systemic venous pressure and lymphatic abnormalities may contribute to disease development. Early recognition and multidisciplinary management are essential to improve clinical outcomes and guide further evaluation of underlying lymphatic abnormalities.

## Introduction

Hypoplastic Left Heart Syndrome (HLHS) is a critical congenital heart defect in which the left side of the heart is underdeveloped, leading to systemic perfusion being dependent on the function of one ventricle [1]. To sustain life, three stages of surgical palliation are required: the Norwood operation, the Glenn shunt, and the Fontan procedure, which direct the systemic venous blood directly to the pulmonary circulation. Although these palliative procedures have improved survival rates, many patients face significant morbidities in the long term. One of the most serious morbidities of the Fontan circulation is protein-losing enteropathy (PLE), which affects 5%–15% of patients with Fontan circulation [2]. PLE is caused by the complex interaction of systemic venous pressure, lymphatic system dysfunction, bowel inflammation, and decreased cardiac output.

The clinical manifestation of PLE involves the excessive loss of plasma proteins into the gastrointestinal tract, leading to hypoalbuminemia, edema, ascites, and immunocompromise [3]. Although significant advances in the care of patients with PLE secondary to the Fontan procedure have been achieved, the clinical condition remains difficult to manage and has high mortality rates. This case report presents the clinical presentation of a child with HLHS and suspected PLE secondary to the Fontan procedure.

## Case presentation

A 10-year-old girl with a known history of hypoplastic left heart syndrome presented with progressive abdominal distension and intermittent episodes of hypoalbuminemia. She had undergone staged palliative repair of a functionally single ventricle. Her Norwood operation was done in infancy, followed by a bidirectional Glenn procedure in 2016. Her Fontan completion involved a fenestrated extracardiac conduit. Because of suspicion of proximal Fontan obstructions, she underwent a redo Fontan in September 2022 with an 18 mm Gelsoft graft. She then underwent a cardiac catheterization procedure with balloon dilatation of the left pulmonary artery, stenting of the inferior vena cava Fontan pathway, and coil occlusion of the right and left internal mammary arteries.

Since early 2025, she had been experiencing increasing abdominal distension with intermittent peripheral edema and morning periorbital swelling that resolves throughout the day. Six months prior to her current admission, she had generalized edema that necessitated hospitalization. She had multiple admissions since then for similar symptoms.

Routine laboratory investigations showed severe hypoalbuminemia with a serum albumin level of 2.3 g/dl, and she was admitted to investigate this further. She also complained of a pruritic rash on her thighs but denied any symptoms of fever, respiratory symptoms, diarrhea, or joint pain.

She had been on furosemide, spironolactone, rivaroxaban, sildenafil, and oral budesonide. She had also required repeated intravenous albumin infusions due to her hypoalbuminemia. She was stable and had a BP of 100/60 mmHg, a heart rate of 100 beats/min, a temp of 37.2°C, and an oxygen saturation of 99% on room air. Her physical examination revealed grade II digital clubbing and abdominal distension with a well-healed surgical scar in the midline. Her liver was palpable 4 cm below the right costal margin. No shifting dullness was present. Her cardiovascular examination revealed a regular rhythm without any murmurs, and her lung examination revealed clear bilateral air entry. Laboratory tests were consistent with hypoalbuminemia (2.3 g/dl). The patient had normal levels of hemoglobin (12.1 g/dl), white blood cells (7.3 × 10^9^/l), and platelets (453 × 10^9^/l). The electrolytes were within normal limits. The liver function tests were also within normal limits. The coagulation tests showed a prothrombin time of 16.2 seconds and an INR of 1.28.

Ultrasonography showed mild free fluid in the subhepatic and pelvic spaces. Echocardiogram showed normal ventricular function with patent Glenn and Fontan circuits and laminar flow. The right-to-left shunt through the Fontan fenestration was also observed. Cardiac catheterization showed a patent Fontan circuit with normal pulmonary artery pressures and pulmonary vascular resistance. However, the inferior vena cava pressure was approximately 16 mmHg, representing mildly elevated systemic venous pressure despite the absence of significant anatomical obstruction.

Based on clinical presentation and exclusion of other causes of hypoalbuminemia, protein-losing enteropathy was considered. Differential diagnoses including nephrotic syndrome, chronic liver disease, malnutrition, and other causes of gastrointestinal protein loss such as intestinal lymphangiectasia and inflammatory bowel disease were considered and excluded based on clinical evaluation, laboratory findings, and the absence of significant proteinuria or abnormal liver function tests.

The management included IV albumin infusions for correction of severe hypoalbuminemia and continuation of diuretic therapy with furosemide and spironolactone for fluid control. Anticoagulation with rivaroxaban was continued due to the risk of thrombosis in patients with Fontan circulation. Pulmonary vasodilator therapy with sildenafil was continued to optimize pulmonary vascular resistance.

Oral budesonide therapy was initiated to decrease inflammation in the intestine and reduce protein loss. Further evaluation with lymphatic imaging was planned to evaluate possible lymphatic abnormalities and to assess whether lymphatic embolization and further Fontan procedures would be needed.

## Discussion

Protein-losing enteropathy (PLE) is known to be a late complication of the Fontan circulation and is associated with considerable morbidity and mortality. PLE is known to occur in 5%–15% of patients undergoing the Fontan procedure and is considered one of the most difficult complications of single-ventricle physiology [4].

The pathophysiology of PLE in patients with the Fontan circulation is known to be multi-factorial. Increased systemic venous pressure, reduced cardiac output, and abnormal lymphatic drainage contribute to lymphatic congestion in the intestines and leakage of protein-rich lymphatic fluid into the gastrointestinal tract. Although PLE may occur in patients without significant Fontan circuit obstruction, elevated systemic venous pressures remain an important contributor to its pathogenesis. Increased Fontan pressure can impair lymphatic drainage and promote intestinal lymphatic congestion, resulting in protein loss into the gastrointestinal tract. In the present case, the Fontan circuit was patent and pulmonary hemodynamics were favorable; however, the inferior vena cava pressure was approximately 16 mmHg, suggesting that elevated venous pressure may have contributed to the development of PLE alongside possible lymphatic abnormalities [5].

Clinically, patients may present with peripheral edema, ascites, diarrhea, fatigue, or unexplained hypoalbuminemia. The diagnosis is usually made by identifying hypoalbuminemia without other causes of hypoalbuminemia such as nephrotic syndrome, liver disease, or nutritional deficiencies. In addition, excessive intestinal protein loss may be confirmed by measuring stool alpha-1 antitrypsin clearance [3].

Several conditions should be considered in the differential diagnosis of hypoalbuminemia and edema in patients with Fontan circulation. Nephrotic syndrome is an important consideration and is typically characterized by significant proteinuria, hypoalbuminemia, and edema. Chronic liver disease may also cause hypoalbuminemia due to impaired hepatic protein synthesis. In addition, malnutrition and malabsorption disorders can lead to decreased serum albumin levels. Gastrointestinal causes of protein loss such as intestinal lymphangiectasia or inflammatory bowel disease should also be considered. In the present case, the absence of significant proteinuria, normal liver function tests, and lack of gastrointestinal inflammatory symptoms made these diagnoses less likely, supporting the diagnosis of protein-losing enteropathy associated with Fontan physiology.

Management of patients with Fontan PLE has been directed at improving Fontan circuit hemodynamics and reducing intestinal protein loss. Medical therapy has included diuretic therapy to manage fluid overload, anticoagulation therapy to prevent thrombosis, and pulmonary vasodilators to improve pulmonary vascular resistance. Corticosteroids, especially oral budesonide, have increasingly been used to treat PLE. Budesonide has localized anti-inflammatory effects on the GI tract. Several studies have documented an increase in serum albumin and resolution of symptoms after oral budesonide therapy. However, there is considerable variability in response to therapy.

A summary of the previously published cases of Fontan PLE compared to the current case is provided in Table 1. From the literature, it is evident that oral budesonide therapy has been associated with an increase in albumin level in various cohorts of patients with PLE, including case reports and systematic reviews [6–9]. Again, however, there is considerable variability in response to therapy.

**Table 1:** Comparison of previously reported cases of Fontan-associated protein-losing enteropathy and the present case.

Study	Population	Albumin (g/dl)	Treatment	Outcome
Thacker et al., 2010 [6]	9 Fontan patients with PLE	1.9	Oral budesonide	Albumin improved to 2.9 g/dl
Schumacher et al., 2011 [7]	10 Fontan patients	Variable	Budesonide therapy	Symptomatic improvement in ~ 80%
Gursu et al., 2014 [8]	4 pediatric Fontan patients	2.25	Budesonide	Albumin increased to 4.15 g/dl
Kewcharoen et al., 2020 [9]	Systematic review (36 patients)	Variable	Budesonide	Mean albumin increase +1.28 g/dl
Montanaro et al., 2024 [10]	Fontan patient with new-onset PLE	Severe hypoalbuminemia	Medical therapy	Clinical stabilization
Midodrine case report, 2025 [11]	Adult Fontan failure	Severe	Midodrine	Albumin normalization before transplant
Present case	10-year-old with HLHS	2.3	Albumin + diuretics + sildenafil + budesonide	Ongoing evaluation

Additional therapeutic modalities have been investigated over the years to treat refractory cases of PLE. These modalities include lymphatic imaging and interventional procedures such as lymphatic embolization and pharmacotherapies such as midodrine to improve systemic vascular tone and alleviate venous congestion symptoms [10, 11]. In severe cases of PLE with refractory symptoms to all treatments, heart transplant may be considered as a final solution.

The case described above demonstrates that severe hypoalbuminemia may occur despite a patent Fontan circuit without significant anatomical obstruction. However, the patient’s elevated inferior vena cava pressure (16 mmHg) may have contributed to lymphatic congestion and the development of PLE. This case highlights the multifactorial pathophysiology of PLE, in which elevated systemic venous pressure and lymphatic dysfunction likely interact to produce clinical disease.

## Conclusion

Protein-losing enteropathy remains a challenging long-term complication of the Fontan circulation and may develop even in the absence of significant Fontan circuit obstruction, particularly when elevated systemic venous pressure and lymphatic dysfunction coexist. This case emphasizes the importance of considering PLE in patients with Fontan physiology who present with persistent hypoalbuminemia and edema. Early diagnosis, exclusion of alternative causes of hypoalbuminemia, and prompt initiation of supportive and targeted therapies including corticosteroids such as budesonide are crucial for symptom control. Further evaluation of lymphatic abnormalities may play an important role in guiding advanced therapeutic interventions. Continued research is needed to better understand the pathophysiology and optimize management strategies for this complex condition.

## Data Availability

The data supporting the findings of this case report are contained within the article. Additional details are available from the corresponding author upon reasonable request.

## References

[1] Feinstein JA, Benson DW, Dubin AM. et al. Hypoplastic left heart syndrome: current considerations and expectations. J Am Coll Cardiol 2012;59:S1–42. 10.1016/j.jacc.2011.09.022 22192720 PMC6110391

[2] Mertens L, Hagler DJ, Sauer U. et al. Protein-losing enteropathy after the Fontan operation: an international multicenter study. PLE study group. J Thorac Cardiovasc Surg 1998;115:1063–73. 10.1016/s0022-5223(98)70406-4 9605076

[3] Patel JK, Loomes KM, Goldberg DJ. et al. Early impact of Fontan operation on enteric protein loss. Ann Thorac Surg 2016;101:1025–30. 10.1016/j.athoracsur.2015.09.036 26652137 PMC4874174

[4] Alsaied T, Lubert AM, Goldberg DJ. et al. Protein losing enteropathy after the Fontan operation. Int J Cardiol Congenit Heart Dis 2022;7:100338. 10.1016/j.ijcchd.2022.100338 39712273 PMC11657892

[5] Itkin M, Piccoli DA, Nadolski G. et al. Protein-Losing enteropathy in patients with congenital heart disease. JACC 2017;69:2929–37. 10.1016/j.jacc.2017.04.023 28619193

[6] Thacker D, Patel A, Dodds K. et al. Use of oral budesonide in the management of protein-losing enteropathy after the Fontan operation. Ann Thorac Surg 2010;89:837–42. 10.1016/j.athoracsur.2009.09.063 20172140

[7] Schumacher KR, Cools M, Goldstein BH. et al. Oral budesonide treatment for protein-losing enteropathy in Fontan-palliated patients. Pediatr Cardiol 2011;32:966–71. 10.1007/s00246-011-0029-2 21660539

[8] Gursu HA, Erdogan I, Varan B. et al. Oral budesonide as a therapy for protein-losing enteropathy in children after the Fontan operation. J Card Surg 2014;29:712–6. 10.1111/jocs.12355 24889258

[9] Kewcharoen J, Mekraksakit P, Limpruttidham N. et al. Budesonide for protein losing enteropathy in patients with Fontan circulation: a systematic review and meta-analysis. World J Pediatr Congenit Heart Surg 2020;11:85–91. 10.1177/2150135119872196 31835979

[10] Kacar P, Iannaccone G, McDonnell E. et al. New onset of protein-losing enteropathy in a patient with Fontan circulation after COVID-19 vaccination: dread the cure or the disease? Cardiol Young 2025;35:418–20. 10.1017/S1047951125000204 39865852

[11] Conte SM, Tardo D, Wiltshire S. et al. A case report of midodrine to treat protein-losing enteropathy for heart transplant candidacy. Eur Heart J Case Rep 2025;9:ytaf544. 10.1093/ehjcr/ytaf544 41306472 PMC12644453

